# Mesalazine granule formulation improves clinical data in Crohn's disease compared with tablet formulation

**DOI:** 10.1038/s41598-020-78603-9

**Published:** 2020-12-07

**Authors:** Satoshi Tamura, Natsuki Ishida, Takahiro Miyazu, Shunya Onoue, Shinya Tani, Mihoko Yamade, Yasushi Hamaya, Moriya Iwaizumi, Satoshi Osawa, Takahisa Furuta, Ken Sugimoto

**Affiliations:** 1 First Department of Medicine, Hamamatsu University School of Medicine, 1-20-1 Handayama, Higashi-ku, Hamamatsu, 431-3192 Japan; 2 Department of Endoscopic and Photodynamic Medicine, Hamamatsu University School of Medicine, 1-20-1 Handayama, Higashi-ku, Hamamatsu, 431-3192 Japan; 3 Department of Laboratory Medicine, Hamamatsu University School of Medicine, 1-20-1 Handayama, Higashi-ku, Hamamatsu, 431-3192 Japan; 4 Center for Clinical Research, Hamamatsu University School of Medicine, 1-20-1 Handayama, Higashi-ku, Hamamatsu, 431-3192 Japan

**Keywords:** Crohn's disease, Inflammatory bowel disease

## Abstract

The efficacy of sustained-release preparations of mesalazine as a remission maintenance treatment for Crohn's disease remains to be established. We aimed to examine the changes in compliance rate and clinical data 2 years after switching from mesalazine tablet to granule formulation at our facility among patients with Crohn's disease in remission. We investigated the rate of continuous treatment of mesalazine granules and examined the changes in Crohn’s Disease Activity Index (CDAI) and serum C-reactive protein (CRP), albumin, and hemoglobin (Hb) levels 2 years after the switch. Compliance rate (continuous treatment vs. additional treatment) and continuous treatment rate [good (rate of ≥ 70%) vs. poor (rate < 70%)] were investigated. Of 46 patients, 12 (27.3%) received additional treatment and 32 (72.7%) did not require additional treatment in 2 years. No significant change in CDAI after switching to granule modification was noted in 32 patients in the continuous treatment group. Nevertheless, clinical remission was maintained for 2 years, and serum CRP levels decreased significantly (*P* = 0.023) and Hb levels increased significantly (*P* = 0.002). No change in the compliance rate was found. Our results suggest that mesalazine granule formulation may have a remission maintenance effect that is superior to that of mesalazine tablets.

## Introduction

Crohn's disease (CD) is a refractory chronic inflammatory disease characterized by discontinuous full-thickness granulomatous inflammation and fistulas that could occur anywhere in the digestive tract^1^. Recently, new drugs, such as anti-tumor necrosis factor (TNF)-α antibodies, have been developed and the number of treatment options is increasing. However, there are few drugs that could be used for long-term maintenance of remission. Mesalazine formulations are often used in mild to moderate CD cases, and oral administration of mesalazine at 3.0 g/day to patients with CD is approved by medical insurances in Japan. However, negative reports regarding the effect of mesalazine on CD have bee reported^2–4^, and apparently, a number of doctors routinely use mesalazine in patients with CD without any clear evidence. The European Crohn's and Colitis Organization guidelines^5^ only allow the continued use of mesalazine in low-risk patients with mild colorectal CD who have been in remission, and do not recommend its active use. However, a pooled analysis of nine randomized-controlled trials suggested that the remission-maintaining effect of mesalazine in patients after remission induction by surgical treatment is somewhat significant in suppressing relapse compared with placebo^6^.

While continuous mesalazine administration is essential for obtaining a beneficial therapeutic effect, adherence remains an important issue. Previous reports showed that 30–50% of patients with chronic diseases have low medication adherence rate^7,8^ and that the medication adherence rate among adolescents with inflammatory bowel disease (IBD) has decreased. A previous study demonstrated that high adherence may contribute to improved IBD outcomes^9^.

Mesalazine tablets were mostly used in Japan. In 2015, PENTASA Granules 94%, which is a high-capacity granule formulation, was released. As the new formulation could reduce the number of tablets that need to be administered, improved adherence was expected, which may in turn have a positive effect on CD. However, the long-term effects of mesalazine granule formulations in patients with CD have not been reported.

In our facility, mesalazine formulation was completely changed from tablets (PENTASA tablets) to granules (PENTASA Granules 94%) since January 2016. Thus, this study aimed to investigate whether the disease state changed after changing the drug formulation in patients with CD, specifically in relation to maintenance of remission. We prospectively examined changes in clinical data 2 years after the switch from mesalazine tablets to granules.

## Results

### Background of enrolled patients

**Figure 1:**
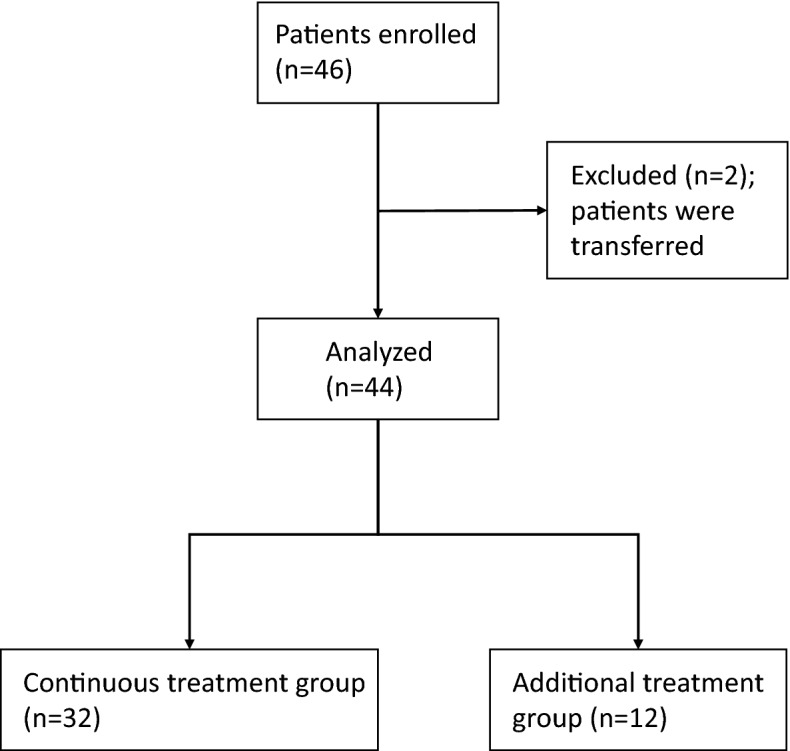
Research design summary and treatment results.

Of the 46 patients with CD enrolled in this study, two were excluded because they were transferred to a different hospital and thus could not be followed up. Hence, statistical analysis was performed in 44 patients (Fig. 1). Data of 44 patients who received mesalazine granules are as follows: average age 39.9 ± 13.0 years, 29 males and 15 females, average disease duration 12.9 ± 9.1 years, and average duration of mesalazine tablet use before switching to mesalazine granules 11.7 ± 7.4 years. The durations of use of therapeutic agents other than mesalazine were 3.9 ± 3.0 years for TNF preparations, 11.6 ± 10.4 years for elemental diets, 15.8 ± 6.3 years for steroids, and 13.1 ± 9.1 years for immunomodulators (data not shown). Twenty patients had anal lesions and 25 had a history of enterectomy surgery. The concomitant medications at the start of the study were immunomodulators (40.9%), steroids (4.5%), anti-TNF (45.5%), and elemental diet (59.1%). There were no cases in which treatment was changed within 6 months before switching to granules. We also reviewed 38 cases whose data could be retrospectively compared 2 years before switching to granules. Then, we compared the changes in the data for Crohn’s Disease Activity Index (CDAI) and serum C-reactive protein (CRP), albumin (Alb), and hemoblogin (Hb) levels for 2 years when the patients were taking 5ASA tablets, but no significant difference was found in any case (Supplemental Table S1). Montreal classification L1, L2, and L3 were observed in 13, 4, and 27 patients, respectively, and the compliance rate was 81.0 ± 23.2% (Table 1). No patient had treatment discontinuation due to adverse events resulting from the formulation change. The compliance rate for mesalazine tablets was 83.3%, which was not significantly different from the compliance rate for mesalazine granules (81.0%; *P* = 0.491) (data not shown).

**Table 1:** Demographic characteristics of the 44 patients with Crohn’s disease.

	Total (N = 44)	Continuous treatment group (n = 32)	Additional treatment group (n = 12)	P*
Age at the start of treatment with mesalazine granule formulation (years)	39.9 ± 13.0	41.0 ± 14.8	37.0 ± 5.6	0.37
Sex (male/female)	29/15	20/12	9/3	0.44
Age at diagnosis (years)	27.0 ± 11.6	27.6 ± 13.1	25.3 ± 6.5	0.56
Disease duration (years)	12.9 ± 9.1	13.4 ± 9.6	11.8 ± 8.2	0.61
Duration of tablet use (years)	11.7 ± 7.4	11.8 ± 7.4	11.3 ± 7.7	0.84
**Smoking history**				
Current/past/never	8/10/26	4/8/20	4/2/6	0.28
Anal lesions (%)	20 (45.5)	10 (31.3)	10 (83.3)	0.002
Surgical history	25 (56.8)	18 (56.3)	7 (58.3)	0.9
**Concomitant drug**				
Immunomodulator	18 (40.9)	14 (43.8)	4 (33.3)	0.53
Steroid	2 (4.5)	1 (3.1)	1 (8.3)	0.46
Anti-TNF	20 (45.5)	16 (50.0)	4 (33.3)	0.32
Elemental diet	26 (59.1)	16 (50.0)	10 (83.3)	0.045
**Montreal classification**				
A1/A2/A3	4/36/4	4/24/4	0/12/0	0.16
L1/L2/L3	13/4/27	10/2/20	3/2/7	0.55
B1/B2/B3	14/17/13	11/10/11	3/7/2	0.24
CDAI	105.5 ± 90.8	101.7 ± 86.9	115.8 ± 101.3	0.65
Serum CRP (mg/dl)	0.34 ± 0.49	0.36 ± 0.52	0.27 ± 0.40	0.61
Alb (g/dl)	4.2 ± 0.5	4.1 ± 0.5	4.4 ± 0.4	0.04
Hb (g/dl)	13.1 ± 1.5	12.9 ± 1.5	13.8 ± 1.5	0.09
Compliance rate (%)	81.0 ± 23.2	80.5 ± 22.0	79.1 ± 19.2	0.81

*TNF* tumor necrosis factor, *CDAI* Crohn’s Disease Activity Index, *CRP* C-reactive protein, *Alb* albumin, *Hb* hemoglobin.

*Continuous treatment group vs. additional treatment group.

### Continuous treatment rate after formulation change

**Figure 2:**
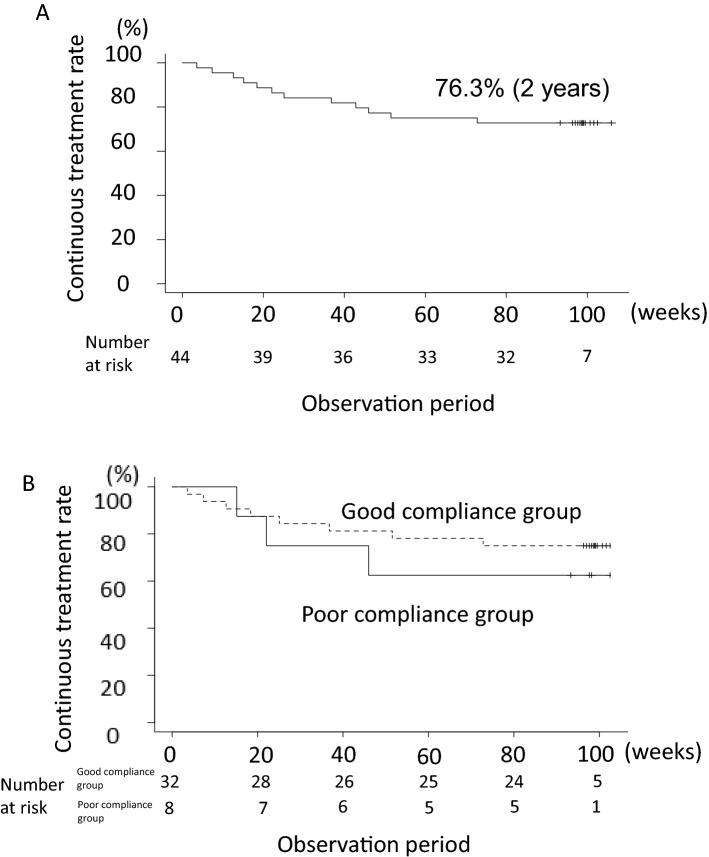
Survival curve of the continuous treatment maintenance rate of mesalazine granule preparation. (**A**) Overall. (**B**) Comparison between the good compliance group (dotted line) and the poor compliance group (solid line).

Twelve of 44 patients (27.3%) needed additional treatment during the 2 years after the switch from mesalazine tablets to granules. Moreover, 32 of 44 (72.7%) remained in remission for 2 years without additional treatment (continuous treatment rate) (Fig. 2A). Additional treatments included induction of anti-TNF in six cases, an increased dose of anti-TNF in three cases, increased dose of immunomodulatory drug in one case, and the addition of budesonide in two cases. The decision to prescribe these additional treatments was left to the attending physician, but most were prescribed because of worsening clinical symptoms and worsening laboratory data. No significant differences in age at the time of change to granule formulation, sex, age at diagnosis, tablet administration duration, smoking history, surgical history, Montreal classification, CDAI, and serum CRP and Hb levels between the continuous treatment and additional treatment groups were found. However, the rate of anal lesions was 31.3% in the continuous treatment group and 83.3% in the additional treatment group (*P* = 0.002; Table 1). Moreover, the compliance rate in the continuous treatment and additional treatment groups was 80.5% and 79.1%, respectively, showing no significant difference (*P* = 0.81; Table 1). Analysis of 44 patients showed no significant difference in progression-free survival between the good compliance (compliance rate of ≥ 70%) and poor compliance groups (compliance rate < 70%) (*P* = 0.49); however, the proportion of patients requiring additional treatment in the poor compliance group tended to be higher (Fig. 2B). In addition, no significant difference in concomitant drug use, including immunomodulators, steroids, and anti-TNF, was found between the continuous treatment and additional treatment groups; however, use of elemental diet was significantly higher in the additional treatment group (83.3%) than in the continuous treatment group (59.1%) (*P* = 0.045; Table 1). Alb levels at the time of change to granule formulation was within the normal range (4.0–5.1 g/dL) in both the continuous treatment and additional treatment groups, although Alb levels were significantly higher in the latter (Table 1).

In addition, since mesalazine has not been shown to have a positive effect on anal lesions in CD, we excluded 22 patients with anal lesions and evaluated the data of those patients in the same manner; we found significant improvement in CRP and Hb levels (Supplemental Fig. S1). We also compared CDAI and levels of CRP, Alb, and Hb in 25 patients who were in clinical remission for more than 1 year, but none of them was significantly different (data not shown).

### Changes in clinical activity and blood test data

**Figure 3:**
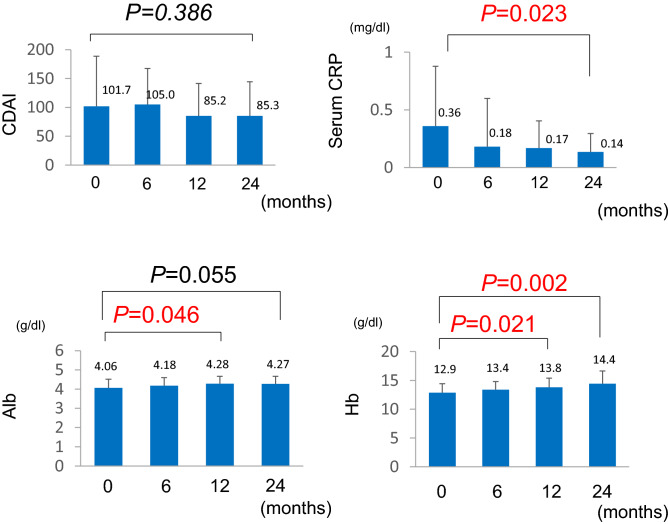
Two-year changes in CDAI and levels of CRP, Alb, and Hb in the continuous treatment group after changing to mesalazine granules. *CDAI* Crohn’s Disease Activity Index, *CRP* C-reactive protein, *Alb* albumin, *Hb* hemoglobin.

We investigated the changes in CDAI and serum CRP, Alb, and Hb levels in the continuous treatment group at 6 months, 1 year, and 2 years after changing from a tablet to granule formulation. CDAI decreased from 101.3 to 85.7 2 years after the tablet was changed to the granule formulation; nonetheless, although a downward trend was noted, no significant change was observed (Fig. 3). Moreover, CDAI levels remained below < 150 throughout the observation period. Furthermore, serum CRP levels decreased (0.36 mg/dl to 0.14 mg/dl; *P* = 0.023), whereas Hb levels increased (12.9 g/dl to 14.4 g/dl; *P* = 0.002), indicating a significant improvement over 2 years (Fig. 3). The Alb level after 2 years was 4.27 g/dl (P = 0.055); although no significant difference was found, the Alb levels tended to improve (Fig. 3). No significant changes in CDAI and serum CRP at 6 months and 1 year after the tablet was changed were observed, whereas a significant improvement in Alb and Hb levels was observed after 1 year [4.28 g/dl (*P* = 0.046) and 13.8 g/dl (*P* = 0.002), respectively; Fig. 3].

### Changes in clinical data in the good compliance group

**Figure 4:**
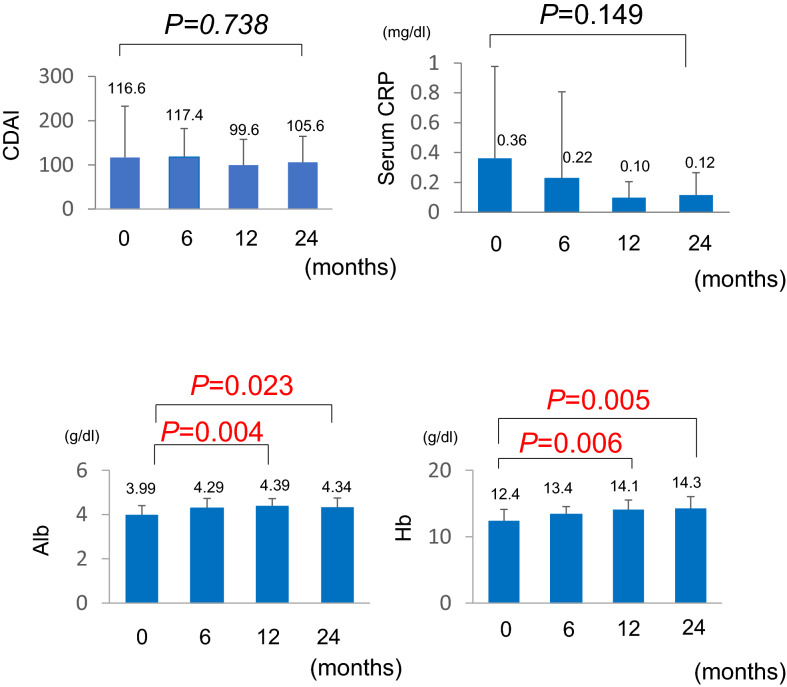
Two-year changes in CDAI and levels of CRP, Alb, and Hb in the continuous treatment group with good compliance after changing to mesalazine granules. *CDAI* Crohn’s Disease Activity Index, *CRP* C-reactive protein, *Alb* albumin, *Hb* hemoglobin.

To clarify whether the compliance rate affected the clinical course in the continuous treatment group, we investigated the changes in CDAI and serum CRP, Alb, and Hb levels in 21 patients in the good compliance group at 6 months, 1 year, and 2 years after changing the tablet formulation to granules. We found that 2 years after the formulation change, CDAI and Alb levels did not change significantly whereas serum CRP and Hb levels showed a significant improvement (Fig. 4). There were only five cases with compliance < 70%. In this group as well, changes in CDAI and serum CRP, Alb, and Hb levels over 2 years were examined, but no significant changes were observed in any variable (Supplemental Table S2, Supplemental Fig. S2).

## Discussion

In this study, we showed that after switching from mesalazine tablets to granules as maintenance medication for CD, 72.7% of the patients did not require additional treatment. In addition, a comparison between the good compliance and poor compliance groups revealed no significant difference in the proportion of patients requiring additional treatment, although the proportion tended to increase in the poor compliance group. Furthermore, in patients who had continuous treatment, serum CRP and Hb levels significantly improved 2 years after the change; Alb levels significantly improved 1 year after the change.

Compliance with mesalazine treatment is vital for maintaining remission in patients with ulcerative colitis (UC)^11^. However, oral mesalazine tablets are large and the number of tablets that need to be administered is high. Hence, PENTASA Granules 94% was developed to improve medication adherence, and it has been prescribed in Japan since 2015. With the advancement in manufacturing technology, mesalazine content was increased to 94%, and the dose was significantly reduced. We anticipated that patient adherence to medication would be improved with the new formulation, and thus, mesalazine prescription was completely switched from PENTASA Tablets to PENTASA Granules 94% in January 2016 at our institution.

Previous studies in patients with UC reported that the granules have higher acceptabilities than the tablets^12,13^. However, the effects of mesalazine compliance on the maintenance of remission in patients with CD have not been reported previously. Moreover, there is much debate about the effect of mesalazine on remission maintenance in CD, and the use of biologics for maintaining remission in patients with mild to moderate CD has not been considered medically and economically favorable. However, the usefulness of mesalazine was described in a Cochrane Review in 2011^6^; thus, mesalazine preparations are often used as remission maintenance therapy for CD in Japan. In our study, we showed that high compliance to mesalazine treatment could also result in high remission maintenance rates among patients with CD.

In this study, the compliance rate for oral mesalazine tablets was 83.3%, which was not significantly different from the compliance rate for mesalazine granules (81.0%). Thus, the compliance rate was not associated with the improvement in CDAI and blood test data 2 years after the change to granule formulation. In this study, a questionnaire was conducted to determine whether patients preferred granules or tablets. Thirteen patients preferred tablets, 11 patients preferred granules, and eight patients had similar preferences, but there was no difference in medication adherence or data improvement between these three groups. We could not identify any factors that improved the clinical activity and blood test data in this study other than the change from PENTASA tablets to PENTASA Granules 94%. However, whether granules are scientifically superior to tablets in terms of pharmacokinetics, i.e., drug accessibility and absorbability in small and large intestine lesions, remains unclear. Nevertheless, a previous study reported that mesalazine suppresses Th1 cell differentiation in a dose-dependent manner in vitro^14^. Furthermore, whether the effects on the systemic immune system differ between tablets and granules is currently unknown; hence, future research to further elucidate the pharmacological mechanisms of the formulations is needed.

Moreover, the proportion of patients with anal lesions and that of patients receiving elemental diet was significantly higher in the additional treatment group than in the continuous treatment group (*P* = 0.002 and *P* = 0.045, respectively). Patients with anal lesions have been reported to have high CD disease activity^15–17^; thus, the additional treatment group in this study possibly included a number of patients with high disease activity. In addition, the reason for the large number of patients receiving an elemental diet in the additional treatment group was unclear, and it was unlikely that the elemental diet itself was the cause of the need for additional treatment. Previous reports demonstrated that the use of elemental diet could reduce the disease activity in CD^18,19^. In our study, analysis of the patients receiving elemental diet in the continuous treatment group (n = 16) showed no significant changes in CDAI and serum CRP 2 years after the change to granule formulation; however, a significant improvement in Alb and Hb levels at 1 year and 2 years after the change, respectively, was observed. As the elemental diet started before the formulation change and the amount was not changed, it was highly likely that only the change in formulation was directly involved in the data improvement.

This study has some limitations. First, this study included a small number of patients from a single institution. Second, the condition of mucosal lesions was unknown; endoscopic evaluation was not performed before and after switching to the granule formulation. Third, the compliance rate was self-reported and thus may not be accurate. Finally, this was a prospective, open observational study and no placebo or PENTASA tablet was used. For future studies, while double-blind trials using placebo may be difficult, a crossover test using tablets seems possible.

In conclusion, this study showed that serum CRP and Hb levels significantly improved after switching from mesalazine tablets to granules and that mesalazine granules could maintain high clinical remission rates over the course of 2 years. While no clear evidence on mesalazine as an effective treatment in maintaining remission in CD has been clearly established, mesalazine granule formulation has a remission maintenance effect that is superior to that of mesalazine tablets, thereby suggesting that the active use of mesalazine granules for remission maintenance in CD may be beneficial. Nonetheless, further basic and clinical studies on the maintenance treatment for CD remission using mesalazine granules are warranted.

## Methods

### Patients

Forty-six patients with CD who were treated at the Hamamatsu University School of Medicine from January 2016 to April 2016 and who switched from sustained-release mesalazine tablets (PENTASA tablets) to granules (PENTASA Granules 94%) were included in this study. All patients provided informed consent prior to enrollment in this study. Patients who did not provide consent were excluded. Patients with other IBD, such as UC, Behcet’s disease, and indeterminate colitis, were also excluded. Laboratory tests, including complete blood count and blood biochemical analysis, and CDAI measurements were performed at baseline, at 6 months and 12 months, and at the end of the study period (24 months).

### Ethical approval

The study protocol was reviewed and approved by the ethics committee of Hamamatsu University School of Medicine (16-268). Further, the investigation was conducted in accordance with Good Clinical Practice principles and in adherence to the Declaration of Helsinki. All patients provided informed consent prior to enrollment in this study.

### Study design

This study is a single center, prospective, observational, open-label pilot study. Two patients who received mesalazine tablets at 1500 mg/day, 3 at 2000 mg/day, and 41 at 3000 mg/day switched to the granule formulation, without changing the amount of mesalazine. Both sustained-release mesalazine tablets and granules were administered twice or three times a day; the dose and frequency of administration were not changed after the formulation was changed. To quantify the compliance rate for mesalazine tablets before the switch, we conducted a questionnaire survey to the patients using a visual analogue scale according to previous reports^10^. At the end of the study period, a questionnaire survey was also performed to determine the compliance rate for mesalazine granules. Mesalazine compliance of ≥ 70% was defined as good compliance and that < 70% as poor compliance. Patients receiving immunomodulators, biologics, or enteral nutrition were eligible for inclusion; however, the dose of concomitant medications could not be changed during the observation period. Moreover, patients with increased drug dosage due to symptom exacerbation during the study period or those who received new additional treatment were included in the additional treatment group; these patients were excluded from laboratory tests and clinical severity analysis. Patients who did not require additional treatment were assigned to the continuous treatment group. Additional treatment for symptom exacerbation was left to the judgment of the attending physician.

### Clinical assessment and trial endpoints

The primary endpoints were continuous treatment rates (i.e., no additional treatment) of the mesalazine granule formulation and changes in CDAI and in serum CRP, Alb, and Hb levels 2 years after the formulation change. The secondary endpoints were changes in CDAI and CRP, Alb, and Hb levels at 6 months and 12 months after the formulation change; compliance rate in the continuous treatment and additional treatment groups; and percentage of patients with good treatment compliance (good compliance group) and those with poor adherence to treatment (poor compliance group).

### Statistical analysis

CDAI and blood test data were statistically analyzed by Friedman test. Mann–Whitney U test was performed to determine the difference in compliance rate between the additional treatment and continuous treatment groups. In addition, the time when additional treatment was required was defined as the exacerbation point, and the progression-free survival time between the good and poor compliance groups was evaluated by the log-rank test. All significant differences were defined as *P* < 0.05. IBM SPSS statistics version 25.0 (SPSS Inc., Chicago, IL, USA) was used for all analyses.

## Supplementary Information

Supplementary Figures.

Supplementary Tables.

## Data Availability

Data available upon request from Ken Sugimoto (sugimken@hama-med.ac.jp).
