# Supplementary material for: Mesalazine granule formulation improves clinical data in Crohn's disease compared with tablet formulation
Source: Sci Rep. 2020 Dec 7;10:21353. doi: 10.1038/s41598-020-78603-9 (PMC7721736; doi:10.1038/s41598-020-78603-9)

Mesalazine granule formulation improves clinical data in Crohn's disease compared with tablet formulation

Satoshi Tamura[1], Natsuki Ishida[1], Takahiro Miyazu[1], Shunya Onoue[1], Shinya Tani[1], Mihoko Yamade[1], Yasushi Hamaya[1], Moriya Iwaizumi[3], Satoshi Osawa[2], Takahisa Furuta[4], and Ken Sugimoto[1*]

[1]First Department of Medicine, Hamamatsu University School of Medicine, 1-20-1 Handayama, Higashi-ku, Hamamatsu 431-3192, Japan

[2]Department of Endoscopic and Photodynamic Medicine, Hamamatsu University School of Medicine, 1-20-1 Handayama, Higashi-ku, Hamamatsu 431-3192, Japan

[3]Department of Laboratory Medicine, Hamamatsu University School of Medicine, 1-20-1 Handayama, Higashi-ku, Hamamatsu 431-3192, Japan

[4]Center for Clinical Research, Hamamatsu University School of Medicine, 1-20-1 Handayama, Higashi-ku, Hamamatsu 431-3192, Japan

***Corresponding author**:

Ken Sugimoto, MD, PhD

First Department of Medicine, Hamamatsu University School of Medicine, 1-20-1 Handayama, Higashi-ku, Hamamatsu 431-3192, Japan

Tel: 81 53 435 2261

Fax: 81 53 434 9447

Email: sugimken@hama-med.ac.jp

Supplemental Figure S1

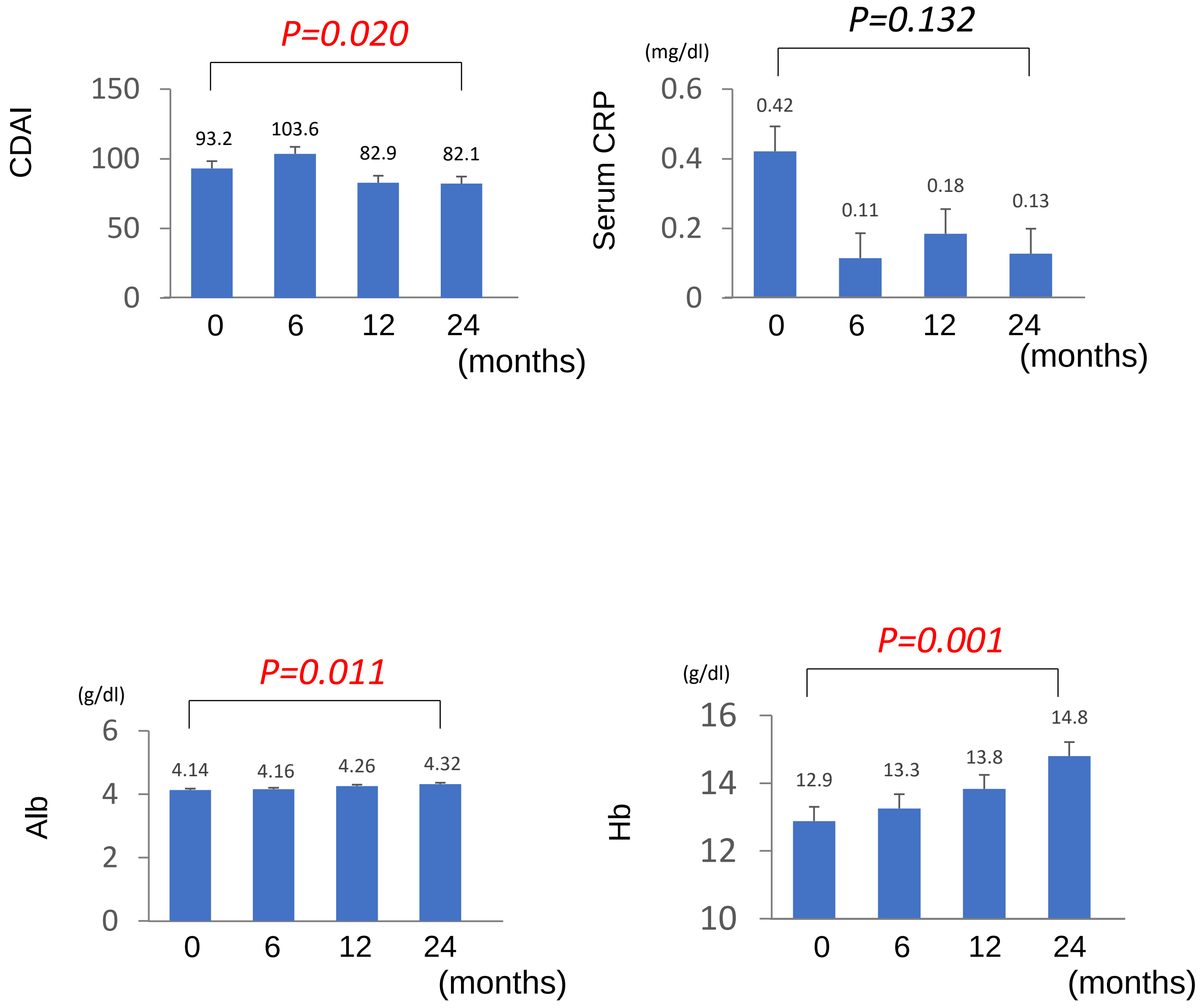

Supplemental Figure S2

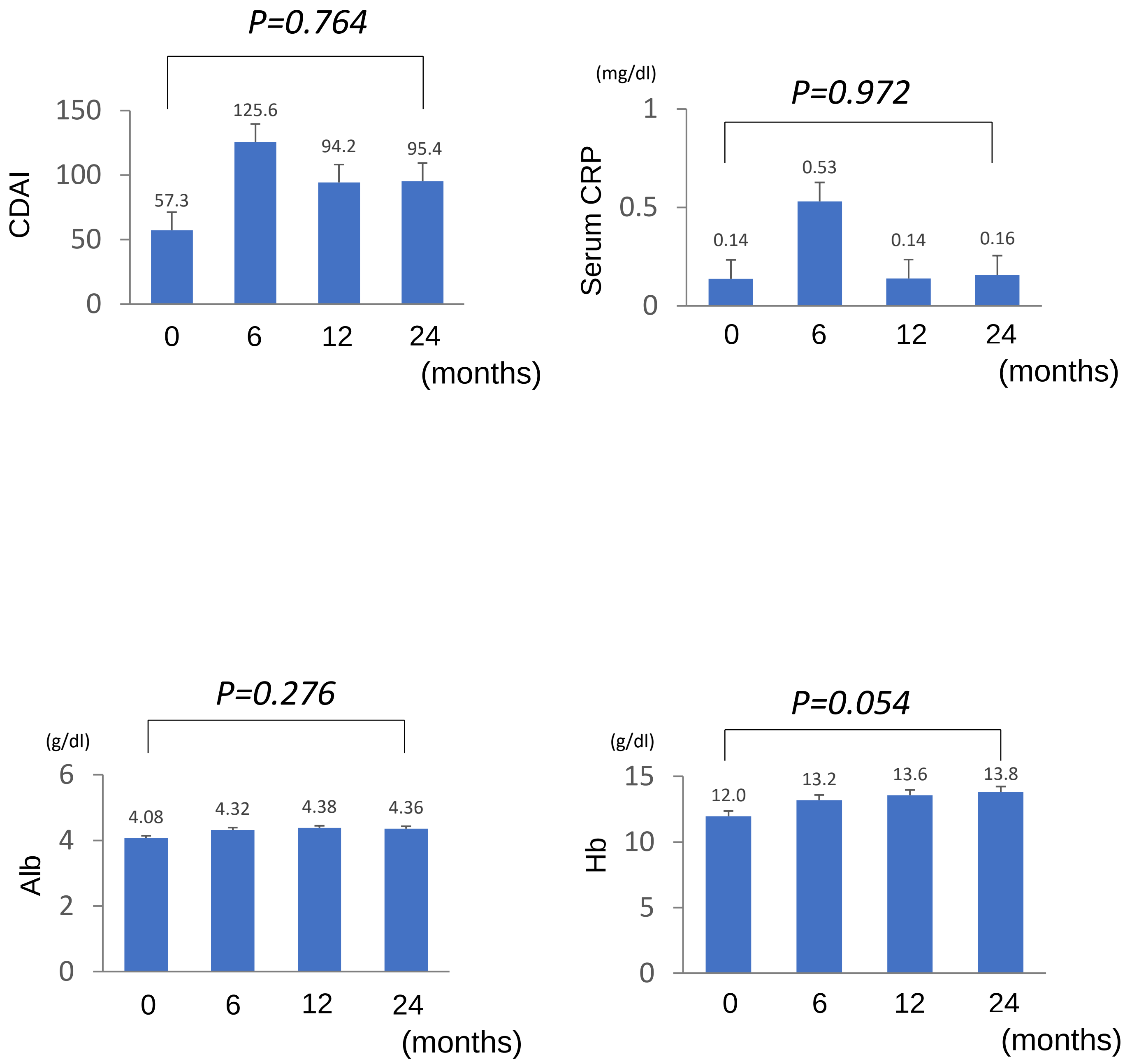

Supplement: Supplementary file 1 — Supplementary Figures. [file 41598_2020_78603_MOESM1_ESM.pdf]
