# Supplementary material for: Mesalazine granule formulation improves clinical data in Crohn's disease compared with tablet formulation
Source: Sci Rep. 2020 Dec 7;10:21353. doi: 10.1038/s41598-020-78603-9 (PMC7721736; doi:10.1038/s41598-020-78603-9)
Supplement: Supplementary file 2 — Supplementary Tables. [file 41598_2020_78603_MOESM2_ESM.docx]

Supplemental Table S1. Comparison of data from two years prior to entry and at the time of entry

|  | Two years prior to entry | At the time of entry | P |
| --- | --- | --- | --- |
| CDAI | 126.5 ± 114.7 | 104.7 ± 87.7 | 0.50 |
| Serum CRP level (mg/dl) | 0.34 ± 0.60 | 0.38 ± 0.53 | 0.93 |
| Alb level (g/dl) | 4.1 ± 0.6 | 4.0 ± 0.5 | 0.66 |
| Hb level (g/dl) | 13.2 ± 1.8 | 12.8 ± 1.5 | 0.67 |

CDAI, Crohn’s Disease Activity Index; CRP, C-reactive protein; Alb, albumin; Hb, hemoglobin

Supplemental Table S2. Demographic characteristics of the 26 patients with Crohn’s disease

|  | Good compliance group  (n = 21) | Poor compliance group (n = 5) |
| --- | --- | --- |
| Age at the start of treatment with mesalazine granule formulation (years) | 13.1 ± 9.8 | 5.3 ± 3.4 |
| Sex (male/female) | 13/8 | 4/1 |
| Age at diagnosis (years) | 28.2 ± 13.4 | 21.6 ± 9.6 |
| Disease duration (years) | 12.9 ± 9.1 | 12.3 ± 8.4 |
| Duration of tablet use (years) | 41.1 ± 12.9 | 34.4 ± 15.1 |
| Smoking history |  |  |
| Current/past/never | 2/6/1 | 1/1/3 |
| Anal lesions (%) | 7(33.3) | 2(40.0) |
| Surgical history | 9(42.9) | 4(75.0) |
| Concomitant drug |  |  |
| Immunomodulator | 8(38.0) | 4 (80.0) |
| Steroid | 1(4.8) | 0(0) |
| Anti-TNF | 12(57.1) | 3 (60.0) |
| Elemental diet | 10(47.6) | 3 (60.0) |
| Montreal classification |  |  |
| A1/A2/A3 | 2/16/3 | 1/4/0 |
| L1/L2/L3 | 7/0/14 | 2/0/3 |
| B1/B2/B3 | 9/5/7 | 1/1/3 |
| CDAI | 119.7 ± 98.8 | 87.2 ± 42.1 |
| Serum CRP level (mg/dl) | 0.35 ± 0.45 | 0.14 ± 0.16 |
| Alb level (g/dl) | 4.1 ± 0.5 | 4.1 ± 0.5 |
| Hb level (g/dl) | 13.1 ± 1.7 | 12.8 ± 1.5 |

TNF, tumor necrosis factor; CDAI, Crohn’s Disease Activity Index; CRP, C-reactive protein; Alb, albumin; Hb, hemoglobin

Supplemental Figure S1. Two-year changes in CDAI and levels of CRP, Alb, and Hb excluding cases with anal lesions after changing to mesalazine granules.

CDAI, Crohn’s Disease Activity Index; CRP, C-reactive protein; Alb, albumin; Hb, hemoglobin

Supplemental Figure S2. Two-year changes in CDAI and levels of CRP, Alb, and Hb in the poor compliance group after changing to mesalazine granules.

CDAI, Crohn’s Disease Activity Index; CRP, C-reactive protein; Alb, albumin; Hb, hemoglobin
